# Discussions of miscarriage and preterm births on Twitter

**DOI:** 10.1111/ppe.12622

**Published:** 2020-01-08

**Authors:** Nina Cesare, Olubusola Oladeji, Kadija Ferryman, Derry Wijaya, Karen D. Hendricks‐Muñoz, Alyssa Ward, Elaine O. Nsoesie

**Affiliations:** ^1^ Department of Global Health School of Public Health Boston University Boston MA USA; ^2^ Department of Technology, Culture, and Society Tandon School of Engineering New York University New York NY USA; ^3^ Department of Computer Science Boston University Boston MA USA; ^4^ Department of Pediatrics Virginia Commonwealth University School of Medicine Richmond VA USA; ^5^ Children's Hospital of Richmond Richmond VA USA

**Keywords:** miscarriage, preterm birth, social media, spontaneous abortion

## Abstract

**Background:**

Experiences typically considered private, such as, miscarriages and preterm births are being discussed publicly on social media and Internet discussion websites. These data can provide timely illustrations of how individuals discuss miscarriages and preterm births, as well as insights into the wellbeing of women who have experienced a miscarriage.

**Objectives:**

To characterise how users discuss the topic of miscarriage and preterm births on Twitter, analyse trends and drivers, and describe the perceived emotional state of women who have experienced a miscarriage.

**Methods:**

We obtained 291 443 Twitter postings on miscarriages and preterm births from January 2017 through December 2018. Latent Dirichlet Allocation (LDA) was used to identify major topics of discussion. We applied time series decomposition methods to assess temporal trends and identify major drivers of discussion. Furthermore, four coders labelled the emotional content of 7282 personal miscarriage disclosure tweets into the following non‐mutually exclusive categories: grief/sadness/depression, anger, relief, isolation, annoyance, and neutral.

**Results:**

Topics in our data fell into eight groups: celebrity disclosures, Michelle Obama's disclosure, politics, healthcare, preterm births, loss and anxiety, flu vaccine and ectopic pregnancies. Political discussions around miscarriages were largely due to a misunderstanding between abortions and miscarriages. Grief and annoyance were the most commonly expressed emotions within the miscarriage self‐disclosures; 50.6% (95% confidence interval [CI] 49.1, 52.2) and 16.2% (95% CI 15.2, 17.3). Postings increased with celebrity disclosures, pharmacists’ refusal of prescribed medications and outrage over the high rate of preterm births in the United States. Miscarriage disclosures by celebrities also led to disclosures by women who had similar experiences.

**Conclusions:**

This study suggests that increase in discussions of miscarriage on social media are associated with several factors, including celebrity disclosures. Additionally, there is a misunderstanding of the potential physical, emotional and psychological impacts on individuals who lose a pregnancy due to a miscarriage.

**Synopsis**

**Study question**

How are miscarriages and preterm births discussed on social media?

**What's already known**

Studies suggest that there is a trend towards expressing disenfranchised grief on social media. However, no large studies have investigated trends and discussions around miscarriages and preterm births on Twitter.

**What this study adds**

First, we show that there are multiple conversation topics related to miscarriages and preterm births. Second, we demonstrate that specific events usually drive surges in discussions. Lastly, in addition to grief, women who have experienced a miscarriage use social media to share feelings towards insensitive comments by clinicians, friends, and family; health care costs; legislatures affecting women's health etc Our findings can inform clinician‐patient communication regarding miscarriages and preterm births.

## 1 INTRODUCTION

Wearables, mobile devices, and social media offer unique opportunities for studying individual‐level health.^1^, ^2^, ^3^, ^4^ Research suggests that these data can be used in the study of sensitive health topics such as suicidal ideation and postpartum depression.^5^, ^6^, ^7^, ^8^, ^9^ Social media have also enabled the public discussion of private or stigmatised topics such as miscarriage—the spontaneous abortion of a viable fetus during the first 20 weeks of pregnancy.^10^ Although an estimated 15%‐20% of known pregnancies end in a miscarriage, data suggest miscarriages are largely misunderstood and those affected can feel isolated.^11^ In fact, in a 2015 survey of 1084 participants which included men and women, 55% believed that 5% or fewer pregnancies end in a miscarriage.^11^

It is also well documented that the loss of a fetus can be traumatic^12^, ^13^, ^14^, ^15^, ^16^, ^17^, ^18^ and that individuals experience feelings of loss, grief, guilt, isolation, and shame.^11^, ^19^, ^20^ Typically, when death occurs, families can grieve and support each other. However, those who experience a miscarriage do not always share their experiences due to many reasons, including incorrect perceptions of how often it occurs, and are more likely to grieve alone.^21^ Also, some women may not feel adequately supported by their partner or health care provider.^22^, ^23^ Women often feel unhappy with the level of information they receive regarding pregnancy complications,^12^, ^23^, ^24^ information about the causes of miscarriages is sometimes withheld for fear of inciting psychological distress 17 and miscarriage continues to be a stigmatised topic.^25^ In addition, follow‐up care post‐miscarriage is usually the patient's responsibility, and though there are some models for care following a miscarriage, screening for psychological distress is not uniformly practised.^21^, ^26^ Tellingly, a recent article in the New England Journal of Medicine outlining better medical management of miscarriage did not include any mention of the possibility of psychological morbidity and the need to screen for mental health.^27^ This lack of information and support might lead some women to seek help from other sources.

Social networks can play a crucial role in helping and encouraging individuals after pregnancy loss by providing a positive support system that helps in lessening feelings of grief and loss.^28^ Social media sites such as Twitter—a social networking site where people post short messages (currently limited to 280 characters) called tweets—may serve as spaces where users feel comfortable sharing highly personal experiences and thoughts—even those related to their medical history.^29^ These platforms may function as contexts in which the disenfranchised grief and desire for information associated with miscarriage may be acknowledged and validated. Pregnant and postpartum women are increasingly using social media to get health information on infant care and self‐care services regarding pregnancy, and also to inform others about their miscarriages and seek support.^30^ Social media might also provide a space for women feeling isolated to discuss their experience and to seek support from a community with similar experiences.^31^ Research suggests that women feel comfortable learning about other women's experiences of miscarriage, and some feel that only those who have also experienced a miscarriage can truly understand the experience.^12^ Furthermore, survey respondents say that disclosures of miscarriage by public figures can make women feel less isolated.^11^

In this paper, we use social media data to assess current public perception and shared experiences of miscarriages and preterm births. First, we characterise general discussions of miscarriage and preterm births on Twitter. Next, we analyse trends in discussion of miscarriage and preterm births discussions on Twitter. Lastly, we characterise the perceived emotional state of women who have experienced a miscarriage and discuss assumed causes.

## 2 METHODS

### 2.1 Topic modelling to identify major topics of discussion

We extracted Twitter (https://twitter.com) postings in English containing the phrase “preterm birth” or “miscarriage” and referring to pregnancy loss from January 2017 through December 2018. To accomplish our study aims, we first applied Latent Dirichlet Allocation (LDA)^32^, ^33^, ^34^, ^35^ to identify major topics in the data. LDA is a data‐driven modelling approach widely used for uncovering latent topics within text corpus. The algorithm assumes that documents are generated from a finite pool of “topics,” which are represented by a series of probability distributions associated with specific terms. Words are assigned weights within particular topics given these assumed probability distributions. The initial topic assignments are updated iteratively based on the prevalence of the words across topics and the distribution of topics within documents. We chose to use the variational expectation‐maximisation (VEM) algorithm for topic selection. The topics are inferred by the expert/analyst based on grouping of terms and tweets containing those terms. For example, a topic group containing terms such as, “fetus, ectopic, group, early, blood, sharing, depression, pregnancy, months, bleeding, cause, test, symptoms, prenatal, pressure, fertility, ended, labour, diagnosis,” might be interpreted by an expert as referring to ectopic pregnancy. This inference can be validated by looking at the data. An exploratory analysis led to the selection of ten topics. These topics were not mutually exclusive since a tweet can be classified into multiple topics. However, dividing the data into more than ten topics led to redundancy and analysing less than eight topics yielded less informative results. We therefore selected to use eight topics instead of 10 by merging topic groups with similar content.

### 2.2 Statistical analysis to understand discussion trends

To understand trends and drivers of discussion, we first decomposed the daily time series to seasonal, trend, and remainder components using a seasonal‐trend decomposition (STL) procedure based on the non‐parametric approach; Locally Weighted Least Squares Regression (LOESS).^36^ LOESS smooths the response variable y (ie volume of tweets) given the independent variable x (ie time) by fitting a low‐degree polynomial at each point in the data using weighted least squares. The nearest neighbour algorithm is used to select data points for each weighted least‐square fit. The STL approach uses piecewise combinations of short‐term medians to estimate the underlying data trend. This approach is more robust compared with using the least square mean approach because that can result in overfitting for long‐term time series data. Next, anomalies (ie outliers) were detected by applying the Generalized Extreme Studentized Deviate (GESD) test to the remainder component to identify major surges in miscarriage and preterm birth discussions.^37^, ^38^, ^39^ The GESD identifies multiple outliers while controlling for type I error. Outliers are identified by computing the maximum absolute difference between the y_i_s minus the mean divided by the standard deviation of the sample. We focused on the top 20 percent of anomalies. The anomaly detection was conducted using the R package, anomalize.^40^, ^41^

### 2.3 Sentiment analysis

Additionally, we searched for the phrases—“my miscarriage,” and “I had a miscarriage”—to identify self‐reported miscarriage experiences within our data set. In total, we identified 3442 and 3840 tweets containing the phrases, “I had a miscarriage” and “my miscarriage,” respectively. Employing a thematic analysis approach, two study team members read a subset of the tweets and created a non‐mutually exclusive list of categories of sentiment (grief/sadness/depression, anger, relief, isolation, annoyance, and neutral) inferred from previous studies.^11^, ^28^ Each tweet was coded by two investigators to determine relevance (ie whether tweets reflect personal miscarriage disclosure) and to classify tweet sentiment. The coded tweets were merged into one file, reviewed by the study team, and discrepancies were discussed and categorised. Results are presented for tweets for which both coders agreed on the same label.

The analysis was implemented in Python^42^ and R.^40^, ^43^

### 2.4 Ethics approval

The data were public so Institutional Review Board (IRB) approval was not required. Even though IRB was not required, we also considered the ethical implications of conducting research using public social media data. Scholars have argued that using public posts from social media can cause harm to users and current best practices call for researchers to consider ethical impacts even when formal institutional review is not required.^44^, ^45^ For this study, in order to mitigate potential harms to users who posted publicly, we did not publish names of users, and also slightly altered the texts of the tweets presented in the manuscript so that it would be harder to identify users.

## 3 RESULTS

Our data consisted of 291 443 Twitter postings on miscarriage and preterm births in English from 138 658 users. These included personal and familial disclosures of miscarriages and preterm births, advocacy for reducing stigma associated with miscarriages, and public responses to various topics related to miscarriages and preterm births.

### 3.1 Major topics of discussion

Our first aim was to identify major topics of discussions in our data. The LDA analysis resulted in clustering of tweets into ten interrelated topics—celebrity disclosures, Olympic Gymnast Shawn Johnson's miscarriage disclosure, Michelle Obama's miscarriage disclosure, politics, health care, preterm births, feelings of loss, feelings of anxiety, flu vaccine, and ectopic pregnancies. We combined the topics “feelings of loss” and “feelings of anxiety” and the topics “celebrity disclosures” and “Olympic Gymnast Shawn Johnson's miscarriage disclosure,” resulting in eight topic groups (see Table 1 for sample tweets). The eight topics were as follows: Michelle Obama (8.4% of tweets), celebrity (23.0%), preterm birth (10.9%), politics (17.6%), loss and anxiety (10.1%), ectopic pregnancy (7.5%), health care (10.7%), and influenza vaccine (11.7%).

**Table 1:** Sample miscarriage and preterm birth tweets from our data

Topic	Tweet examples
Celebrity	One miscarriage was enough for me so can't imagine eight or more #GabrielleUnion is really strong @AndrewDEast sorry to hear about your wife's miscarriage. You two appear to have a strong bond and get through anything, even this
Politics	Replying to @OK_Magazine: Its awful that they classify this as a late miscarriage and therefore there's no birth/death certificate. This law needs to be changed! Replying to @username @Slate: I had a $ 1500 ER bill for my miscarriage. I never hear lib or conservative politicians arguing to help me pay for that
Health care	Replying to @username @username: My wife and I had an experience at a hospital when a socially‐lacking doctor intern told us to have fun trying again following our miscarriage In 2014, I walked into a hospital not knowing if doctors would try at all to save my twin boys born at 22 wk. They wouldnt. Hospitals must be transparent about how they handle preterm birth. Illinois needs bipartisan legislation to force hospitals to be clear #WorldPrematurityDay
Preterm birth	This #PrematurityAwarenessMonth, we must take action to bring down America's rising rates of preterm birth. As maternity care caucus co‐chair, I am committed to bringing #BlanketChange to #MaternityCare policies so we can reduce preterm births and keep mothers and babies healthy! Replying to @username: Unfortunately a preterm birth also makes them prone to allergies such as lactose intolerance. That's when mothers selective but rich nutrition along with #Abbott baby formulas like #Isomil aids the little one in deriving the necessary nutrients #FeedIQChatter Abbott #FeedIQ
Michelle Obama	Former first lady Michelle Obama shares deeply personal moments in her soon‐to‐be‐published memoir, discussing infertility, a miscarriage and accuses then‐ private citizen Donald Trump of jeopardizing her familys safety. @MajorCBS has more cbsn.ws/2DejD2L 10 9 18 1. I admire already admire Michelle Obama 2. Everything she says here is my reason for creating Lyfe's Melody. We care about all kinds of grief, but a miscarriage started it all for me… instagram.com/p/Bp‐mcADF74E/
Loss & anxiety	I'm mourning my 7th loss to #miscarriage this morning. #grief Replying to @username: All three of my miscarriages have been natural miscarriage…It's a long process and it is hell. Every trip to the bathroom is a constant reminder of what you have lost
Flu vaccine	Recent @ICESOntario study showed a link between H1N1 #flu and preterm birth among women with chronic health http://issuesices.on.ca/Newsroom/News- For lingering concerns about flu vaccine and risk of miscarriage, @DrPaulOt outlines the flaws of the CDC article (which he argues shouldn't have been published) & the overlooked risk of not vaccinating during pregnancy: The Pregnancy Vaccine Scare That Should Have Ne Why that CDC study on flu shots during the first trimester should never have been published. thedailybeast.com
Ectopic pregnancy	Replying to @username: I have endometriosis and this is likely my future. I've had an ectopic miscarriage. She's so brave. She's now my freaking idol. Replying to @username: I really wish miscarriage wasn't taboo. It seems as if it's dirty and embarrassing to talk about. I've had two mc and an ectopic. Everyone should talk about it and realize it's normal to grieve and have a support network

We selected two tweets to present for each of the eight topic identified by the Latent Dirichlet Allocation (LDA) topic model.

Celebrities were usually considered strong and brave for publicly discussing infertility struggles and miscarriages. Disclosures by celebrities also led to disclosures by women who had similar experiences. There was substantial response to Michelle Obama's miscarriage disclosure, although her experience was twenty years ago. Topic modelling also revealed the presence of online support groups, although that did not emerge as a major topic.

Political discussions about miscarriages referenced legislations proposed during the study period that would impact miscarriages, such as a law passed by the Texas Legislature in 2017 requiring burial or cremation of foetal tissue associated with miscarriages, ectopic pregnancies, and abortions.^46^

Compared to miscarriages, posting on preterm births did not contain politicised content and were more focused on causes, treatment, prevention, and advocacy towards reducing the prevalence of preterm births in the United States. There were also discussions of potential causes of miscarriages (example: smoking can cause ovulation problems, damage your eggs & increase the risk of a miscarriage. #PregPrep #FertilityFriday #quitsmoking #ttc). One study published in 2017 suggested a potential link between flu vaccines and miscarriages, although the data were inconclusive. Responses to this study included physicians who spoke against the decision to publish and anti‐vaccination proponents, who viewed the study as validation for their cause. A follow‐up study using a larger sample size and specifically aiming to assess the relationship between flu vaccines and miscarriages found no association,^47^ which support current flu vaccine recommendations for pregnant women.

### 3.2 Events associated with tweet volume increase

**Figure 1:**
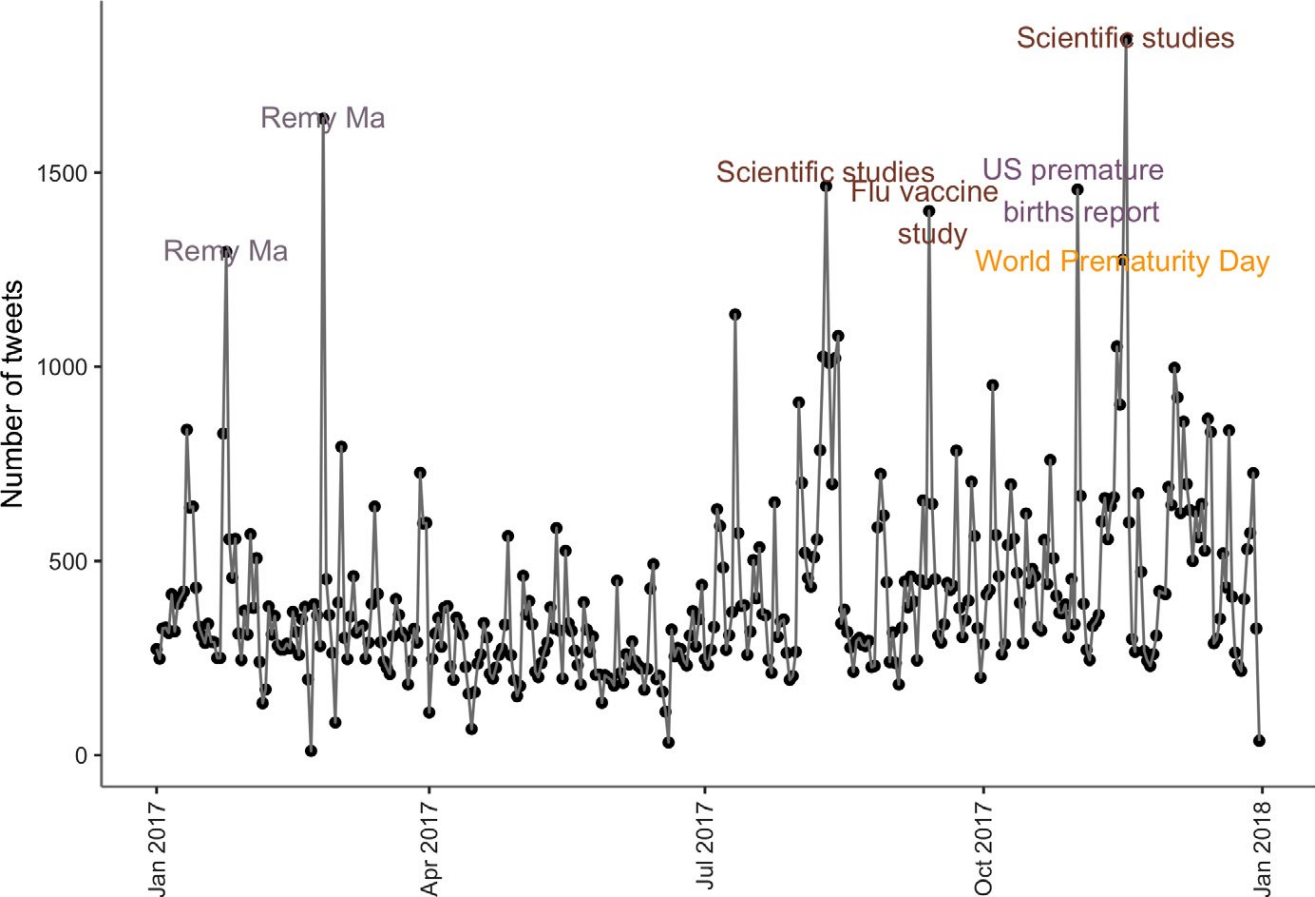
Miscarriage and preterm birth tweets posted in 2017. The labelled dates were detected as anomalies. These included a celebrity disclosure, scientific studies on risks and prevention, world prematurity, and US preterm births report

**Figure 2:**
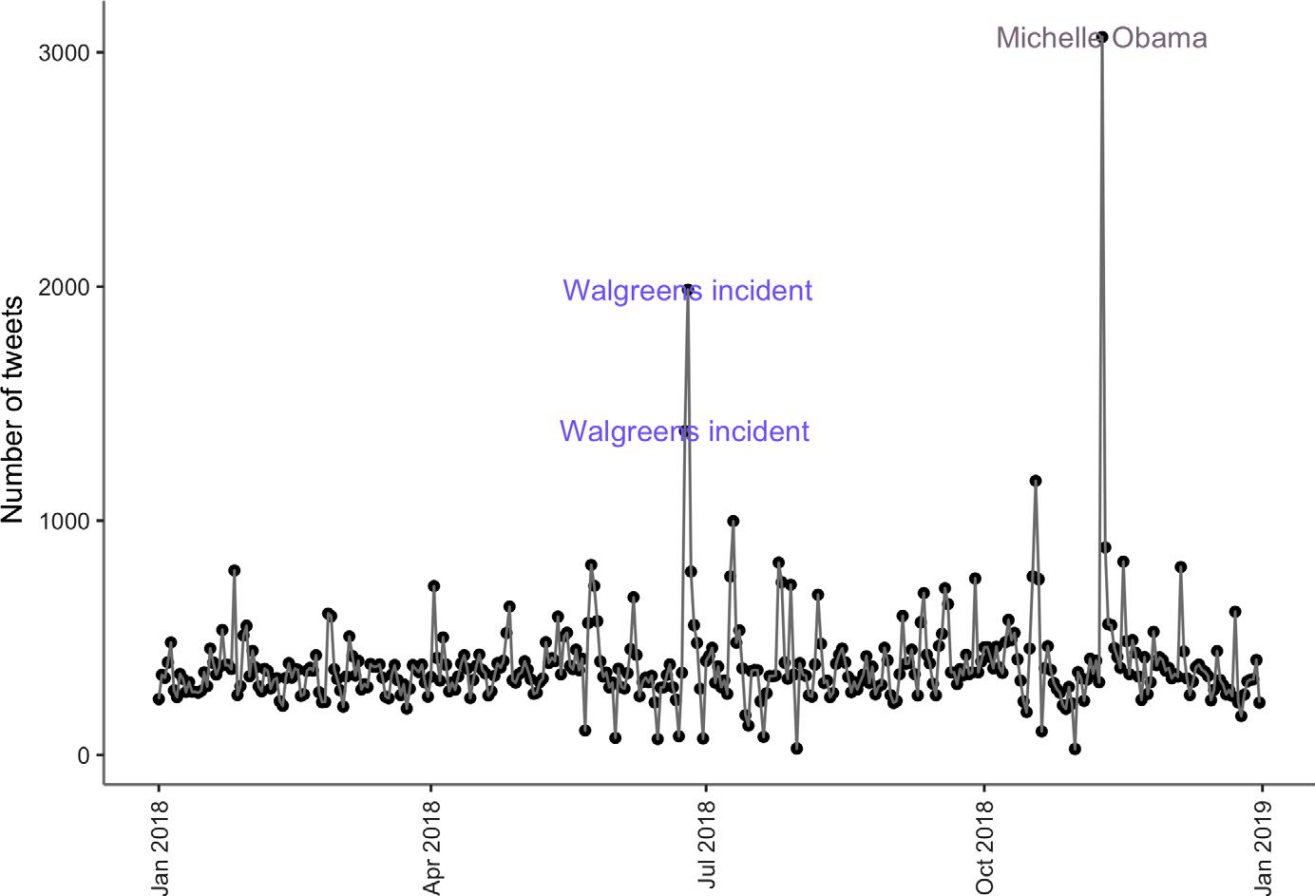
Miscarriage and preterm birth tweets posted in 2018. The labelled dates were detected as anomalies. These included Michelle Obama's disclosure and pharmacist's refusal of prescribed medication to complete a miscarriage

Our second aim was to identify events associated with increases in miscarriage and preterm birth discussions. Our anomaly detection identified ten days with a statistically significant uptake in miscarriage and preterm birth discussions. As shown in Figures 1 and 2, statistically significant increases in discussions of miscarriage that were identified as outliers were associated with celebrity disclosures, research studies on risk factors and preventive methods for miscarriages and preterm births,^48^ reports on preterm birth rates 49 and responses to pharmacists' refusal to administer the medication, Misoprostol, which is commonly used to induce the expulsion of a fetus.^50^ Michelle Obama's miscarriage disclosure had the most significant response, generating 3051 tweets—approximately 3.6 times the magnitude of the highest weekly average.

### 3.3 Personal disclosures of miscarriages

**Figure 3:**
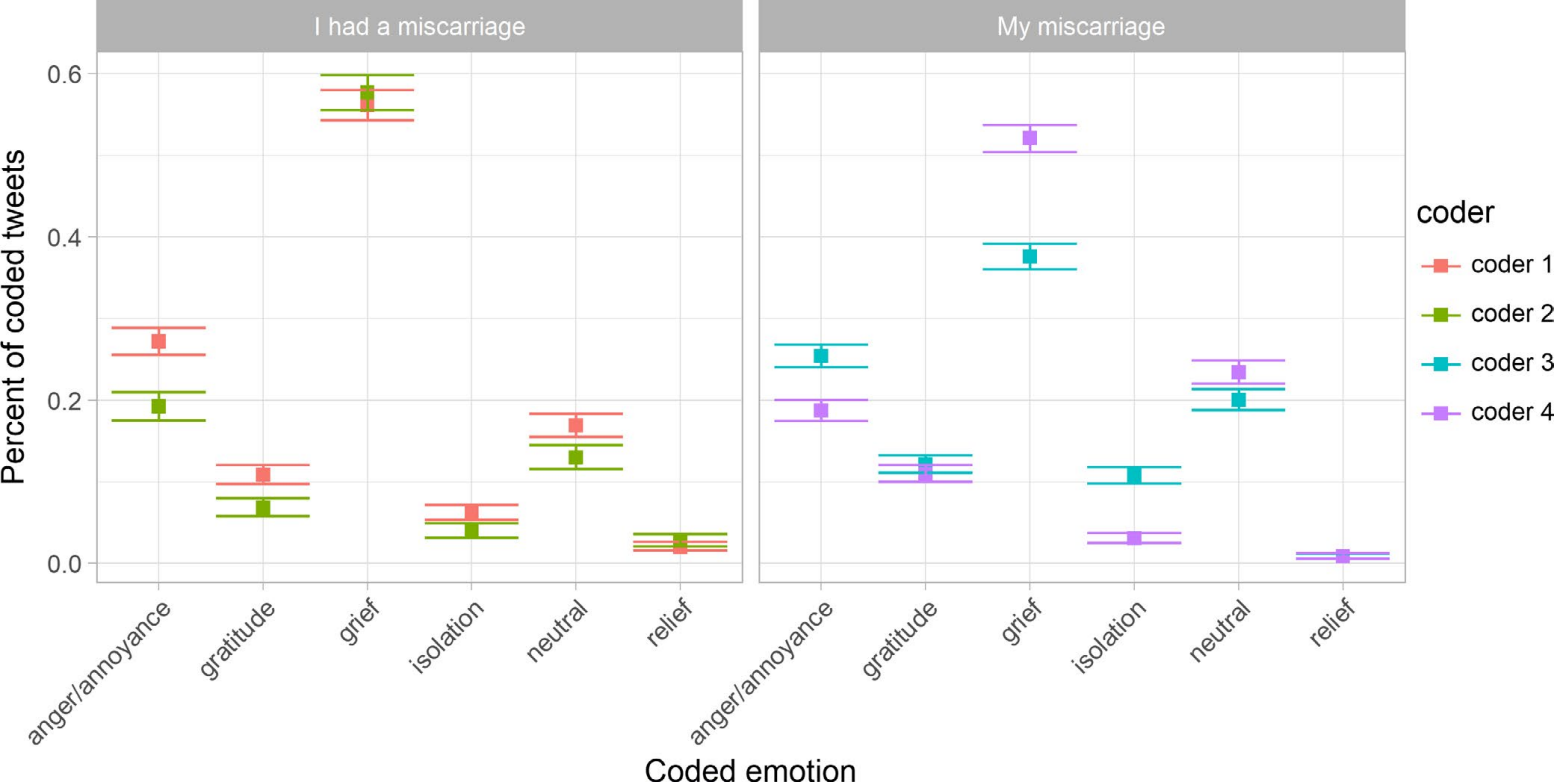
Emotions perceived from tweets on personal miscarriage disclosures. Two coders labelled each tweet

Our third aim was to characterise the emotional state of individuals who self‐reported a miscarriage. The data consisted of 7282 tweets from 5079 users. The emotional labels assigned by the two coders for each data set were similar (Figure 3). See examples of tweets in each emotion category in Table 2. The average F1‐measure^51^ of concordance for “my miscarriage” and “I had a miscarriage” tweet labels were 85.28 and 90.01, respectively.

**Table 2:** Examples of tweets for various sentiments

Emotion	Tweet sentiments
Grief	Today was the toughest day I’ve ever had. I had a miscarriage and my husband and I have not stopped crying. Felt I would share this with all of you. I can tell it is going to take me a while to recuperate…I cannot stop thinking about the reality that I had a miscarriage. I am so emotionally unstable right now
Anger	I had a miscarriage/lost my job and got broken up with, so be silent about your pregnancy/promotion and engagement. What happened to your baby, I thought you was pregnant… I had a miscarriage bitch ya scared assss curious cat
Annoyance	Like when I got fired after I had a miscarriage because I was a liability… and had no job protection ‐ now that was rude! I do not believe in abortion unless rate. Last July, I had a miscarriage. It is not the same. How dare you compare the 2?
Isolation	My miscarriage earlier this year felt so isolating until other mothers and women started talking honestly to me about it we ought to talk about the whole scope of womanhood and not be ashamed of our bodies I had a miscarriage and I have no one to be there for me or even talk to
Relief	"I was 15 and could not sustain a baby. I had a miscarriage before making any appointments" I was 18. I had a miscarriage before making a decision. Now, I can have a happy family because I did not have a baby before
Gratitude	Your channel kept me anchored after I had a miscarriage in June. You have awarded us all so much already. Trust me on this all so much already. Trust me on this Your posts on Instagram have helped me greatly this past week. I had a miscarriage a couple of days ago and it has been so hard…

Among tweets from individuals who self‐reported a miscarriage, approximately 50.6% (95% CI 49.1, 52.2) and 16.2% (95% CI 15.2, 17.3) expressed grief and anger or annoyance, respectively. Many tweets within these categories discussed topics such as insensitive comments from others, or unfair treatment by medical professionals. Tweets also referenced associations between abortion and miscarriage, and noted that this comparison is inaccurate and insensitive to women's experiences.

A faction of tweets—0.6% (95% CI 0.4, 0.8)—expressed relief, typically indicating that they were not personally or circumstantially ready to be a parent. About 14.2% (95% CI 13.2, 15.2) of tweets were neutral, meaning they expressed indifference or had no clear emotional valence.

Some tweets expressed feelings of isolation or desire for support (2.6%, 95% CI 2.20, 3.12). Others expressed feelings of gratitude, typically in response to emotional support, the ability to carry a pregnancy to term after a miscarriage, or towards others for sharing their own miscarriage stories (6.9%, 95% CI 6.3, 7.7).

About 4.7% (95% CI 4.2, 5.4) of tweets blamed their bodies, behaviour, or circumstances for their miscarriage. Commonly cited causes included stress, weight, birth control, physical abuse, genetics, and reproductive disorders, such as polycystic ovary syndrome (PCOS) or endometriosis. These findings suggest an internalisation of their experience as something they could have prevented. Within the data set containing the phrase “I had a miscarriage,” twenty‐one individuals expressed anxiety over the possibility of becoming pregnant again.

## 4 COMMENT

### 4.1 Principal findings

Conversations relating to preterm births were focused on causes, treatment, prevention, and advocacy towards reducing the prevalence of preterm births. In contrast, the conversation on miscarriage was varied and sometimes politicised. However, a large segment of this conversation encouraged individuals to be open about their miscarriage experiences. This suggests that Twitter is not only a place to spread and gather information about miscarriages, it is also a space where users can share personal thoughts and feelings on this topic.

Individuals who reported a miscarriage expressed grief, anger, annoyance, and feelings of isolation. Individuals also believed that a stressful event, or not having enough prenatal care may cause a miscarriage, which may lead to guilt. These feelings of guilt, grief, and isolation were alleviated when those affected received emotional support from family and friends.^11^ Bardos and colleagues^11^ noted that peer support and miscarriage disclosure can help to alleviate the negative emotional effects of a miscarriage. Also, celebrity disclosures of miscarriages were associated with increased interest in miscarriage and personal disclosures of similar experiences. The presence of support group discussions and expression of gratitude towards others for offering support suggest that using Twitter to discuss personal miscarriage experiences may help alleviate feelings of isolation commonly associated with miscarriages.^11^, ^31^

While trends in the data suggest Twitter might be a preferred source for seeking and sharing miscarriage information, the prevalence of false information about causes and treatments of miscarriage has the potential to distort public understanding and contribute to suffering. We noted discussions regarding an unsubstantiated link between miscarriage and vaccines. Research on health misinformation on social media invite further investigation into the content of these discussions, as well as who and what is involved (eg the role of bots and trolls).^52^

### 4.2 Strengths of the study

We used a large sample of 138 658 Twitter users to study public discussions of miscarriages. This included more than 5079 social media users who self‐reported having had a miscarriage. We used interpretable machine learning and statistical modelling approaches to understand major topics and discussion trends. Furthermore, manual labelling of sentiment involved four independent members of the research team and findings were reported based on agreement between labellers. The use of social media and analytics approaches in this study improves understanding of public perception of miscarriages and factors that drive discussions on social media platforms.

### 4.3 Limitations of the data

We acknowledge that this study does not represent all public discourse regarding miscarriage and that combining data from multiple social media platforms may provide a more comprehensive representation of online miscarriage discourse. We anticipate that Twitter, which includes content from individuals, media outlets, celebrities, and other entities, may provide a broad overview of attitudes towards miscarriage. It is possible that conversation from social media sites with reciprocal rather than directed ties will contain more emotionally charged content, or content that reflects social support. Moreover, our sample is limited by the keywords we selected. We found that the term “miscarriage” yields the largest volume of tweets. A Twitter ReST API request for 10 000 tweets containing the term “miscarriage” yields 9951, as opposed to 1987 for “miscarried” and 300 for “miscarrying,” with between 14 and 41 tweets overlapping between these searches. However, the exclusion of these terms might miss segments of the conversation. Additionally, one facet of Twitter data that this study did not address but that future research may consider is the inclusion of key demographic variables in these analyses. Twitter does not explicitly provide variables such as age, race, and gender, but researchers have developed approaches to systematically extract this information from user metadata.^53^, ^54^, ^55^, ^56^, ^57^ Finally, Twitter users do not demographically reflect the population at large. There might be sociodemographic and psychosocial differences between the individuals included in our study and non‐Twitter users. While Twitter offers rich, qualitative insights into users’ experience and opinions, data from Twitter should not be used as a representative substitute for public opinion.

### 4.4 Interpretation

While there are limitations in our study, data from Twitter may be a good source for assessing the experiences and attitudes of diverse populations to identify similarities and compare differences in miscarriage and preterm birth experiences. This is particularly important given racial differences in fertility, preterm births, and maternal health.^58^ Studies also suggest that certain populations may withhold information from clinicians due to a general distrust of the medical community or lack of understanding of the benefits of disclosure.^59^ This suggests that education is critical and needed to combat the stigma associated with miscarriages and to help those who have experienced loss. Health care professionals can contribute immensely in reconstructing the miscarriage experiences and psychological impact faced by many women.^60^ There are limited if any psychological support or resources as part of routine care for grieving parents and women, who experienced pregnancy loss are rarely followed up by health care professionals.^17^, ^60^ Women in numerous studies have recommended that psychological intervention, referral support service, post‐miscarriage follow‐up, increased sensitivity, and awareness should be provided to aid them with their despair.^60^, ^61^ Insights gained from Twitter may help clinicians ask more effective questions to address the topic of miscarriage in a way that fosters communication and trust. Furthermore, our findings can be used by health educators and women support groups.

## 5 CONCLUSIONS

This cross‐sectional analysis of personal and collective discussions regarding miscarriage and preterm births provides important insights into how social media may be used to seek and share health‐related information. Understanding current public perceptions of miscarriage as well as how individuals use Twitter to share information and seek support after miscarriage may help inform the development of clinical educational resources for individuals experiencing miscarriage. In addition, knowing that Twitter is seen by some individuals as a resource after miscarriage may be informative for clinicians who provide miscarriage care and treatment and could potentially be used in clinician‐patient communication and education.

## References

[1] Salathé M , Bengtsson L , Bodnar TJ , et al. Digital epidemiology. PLoS Comput Biol. 2012;8:e1002616. 22844241 10.1371/journal.pcbi.1002616 PMC3406005

[2] Jain SH , Powers BW , Hawkins JB , Brownstein JS . The digital phenotype. Nat Biotechnol. 2015;33:462‐463. 25965751 10.1038/nbt.3223

[3] Prier KW , Smith MS , Giraud‐Carrier C , Hanson CL . Identifying health‐related topics on twitter In: International conference on social computing, behavioral‐cultural modeling, and prediction. Berlin, Germany: Springer; 2011:18‐25.

[4] Nguyen QC , Li D , Meng H‐W , et al. Building a national neighborhood dataset from geotagged Twitter data for indicators of happiness, diet, and physical activity. JMIR Public Health and Surveillance. 2016;2:e158. 27751984 10.2196/publichealth.5869 PMC5088343

[5] De Choudhury M , Counts S , Horvitz EJ , Hoff A . Characterizing and predicting postpartum depression from shared facebook data In: Proceedings of the 17th ACM conference on Computer supported cooperative work & social computing. Ney York, NY: ACM; 2014:626‐638.

[6] De Choudhury M , Counts S , Horvitz E . Predicting postpartum changes in emotion and behavior via social media In: Proceedings of the SIGCHI Conference on Human Factors in Computing Systems. Ney York, NY: ACM; 2013:3267‐3276.

[7] Reece AG , Danforth CM . Instagram photos reveal predictive markers of depression. EPJ Data Science. 2017;6:15.

[8] O'Dea B , Wan S , Batterham PJ , Calear AL , Paris C , Christensen H . Detecting suicidality on twitter. Internet Interv. 2015;2:183‐188.

[9] De Choudhury M , Kiciman E , Dredze M , Coppersmith G , Kumar M . Discovering shifts to suicidal ideation from mental health content in social media In: Proceedings of the 2016 CHI conference on human factors in computing systems. Ney York, NY: ACM; 2016:2098‐2110. 10.1145/2858036.2858207 PMC5659860 29082385

[10] CDC . Facts about Stillbirth. https://www.cdc.gov/ncbddd/stillbirth/facts.html. Accessed November 30, 2019.

[11] Bardos J , Hercz D , Friedenthal J , Missmer SA , Williams Z . A national survey on public perceptions of miscarriage. Obstet Gynecol. 2015;125:1313. 26000502 10.1097/AOG.0000000000000859 PMC4443861

[12] Adolfsson A , Larsson PG , Wijma B , Bertero C . Guilt and emptiness: women's experiences of miscarriage. Health Care Women Int. 2004;25:543‐560. 15354621 10.1080/07399330490444821

[13] Berth H , Puschmann A‐K , Dinkel A , Balck F . The trauma of miscarriage–factors influencing the experience of anxiety after early pregnancy loss. Psychother Psychosom Med Psychol. 2009;59:314‐320. 18821478 10.1055/s-2008-1067540

[14] Day RD , Hooks D . Miscarriage: a special type of family crisis. Fam Relat. 1987;36:305.

[15] Lachmi‐Epstein A , Mazor M , Bashiri A . Psychological and mental aspects and “tender loving care” among women with recurrent pregnancy losses. Harefuah. 2012;151:633‐637, 654. 23367735

[16] Lee C , Slade P . Miscarriage as a traumatic event: a review of the literature and new implications for intervention. J Psychosom Res. 1996;40:235‐244. 8861119 10.1016/0022-3999(95)00579-x

[17] Nikcevic AV , Tinkel SA , Kuczmierczyk AR , Nicolaides KH . Investigation of the cause of miscarriage and its influence on women's psychological distress. Br J Obstet Gynaecol. 1999;106:808‐813. 10453831 10.1111/j.1471-0528.1999.tb08402.x

[18] Séjourné N , Callahan S , Chabrol H . Miscarriage and feelings of guilt: a qualitative study. J Gynecol Obstet Biol Reprod. 2011;40:430‐436. 10.1016/j.jgyn.2011.01.010 21330065

[19] Upton RL . A qualitative study of the experience of miscarriage from patients and providers in the rural US. J Comm Pub Health Nursing. 2019;5:1.

[20] Van P , Meleis AI . Coping with grief after involuntary pregnancy loss: perspectives of African American women. J Obstet Gynecol Neonatal Nurs. 2003;32:28‐39. 10.1177/0884217502239798 12570179

[21] Nynas J , Narang P , Kolikonda MK , Lippmann S . Depression and anxiety following early pregnancy loss: recommendations for primary care providers. Prim Care Companion CNS Disord. 2015;17(1). 10.4088/PCC.14r01721 PMC4468887 26137360

[22] Cecil R . Miscarriage: women's views of care. J Reprod Infant Psychol. 1994;12:21‐29.

[23] Friedman T , Gath D . The psychiatric consequences of spontaneous abortion. British J Psychiatry. 1989;155:810‐813. 10.1192/bjp.155.6.810 2620207

[24] Simmons RK , Singh G , Maconochie N , Doyle P , Green J . Experience of miscarriage in the UK: qualitative findings from the national women's health study. Soc Sci Med. 2006;63:1934‐1946. 16781809 10.1016/j.socscimed.2006.04.024

[25] Bommaraju A , Kavanaugh ML , Hou MY , Bessett D . Situating stigma in stratified reproduction: abortion stigma and miscarriage stigma as barriers to reproductive healthcare. Sex Reprod Healthc. 2016;10:62‐69. 27938875 10.1016/j.srhc.2016.10.008

[26] Jensen KL , Temple‐Smith MJ , Bilardi JE . Health professionals' roles and practices in supporting women experiencing miscarriage: a qualitative study. Aust N Z J Obstet Gynaecol. 2019;59:508‐513. 30338853 10.1111/ajo.12910

[27] Westhoff CL . A better medical regimen for the management of miscarriage. N Engl J Med. 2018;378(23):2232‐2233. 29874544 10.1056/NEJMe1803491

[28] Bellhouse C , Temple‐Smith MJ , Bilardi JE . “It's just one of those things people don't seem to talk about…” women's experiences of social support following miscarriage: a qualitative study. BMC Women's. Health. 2018;18:176. 30373583 10.1186/s12905-018-0672-3 PMC6206670

[29] Rosenkrantz AB , Labib A , Pysarenko K , Prabhu V . What do patients tweet about their mammography experience? Acad Radiol. 2016;23:1367‐1371. 27658329 10.1016/j.acra.2016.07.012

[30] Alqassim MY , Kresnye KC , Siek KA , Wolters MK . Facebook for support versus facebook for research: the case of miscarriage In: Extended abstracts of the 2019 CHI Conference on human factors in computing systems. New York, NY: ACM;2019:p. LBW0216

[31] Nsoesie EO , Cesare N .What the public is saying about miscarriage in 140 characters. https://www.npr.org/sections/health-shots/2017/08/27/542809414/what-the-public-is-saying-about-miscarriage-in-140-characters. Accessed November 30, 2019.

[32] Blei DM , Ng AY , Jordan MI . Latent dirichlet allocation. J Mach Learn Res. 2003;3:993‐1022.

[33] Pritchard JK , Stephens M , Donnelly P . Inference of population structure using multilocus genotype data. Genetics. 2000;155:945‐959. 10835412 10.1093/genetics/155.2.945 PMC1461096

[34] Griffiths TL , Steyvers M . Finding scientific topics. Proc Natl Acad Sci. 2004;101:5228‐5235. 14872004 10.1073/pnas.0307752101 PMC387300

[35] Jagarlamudi J , Daumé H III , Udupa R . Incorporating lexical priors into topic models Proceedings of the 13th Conference of the European chapter of the association for computational linguistics. Stroudsburg, PA: Association for Computational Linguistics;2012:204‐213.

[36] Cleveland RB , Cleveland WS , McRae JE , Terpenning I . STL: a seasonal‐trend decomposition. J Off Stat. 1990;6:3‐73.

[37] Vallis O , Hochenbaum J , Kejariwal A . A novel technique for long‐term anomaly detection in the cloud In: 6th USENIX Workshop on hot topics in cloud computing (hotcloud 14). Philadelphia, PA: USENIX Association; 2014.

[38] Rosner B . On the detection of many outliers. Technometrics. 1975;17:221‐227.

[39] Rosner B . Percentage points for a generalized ESD many‐outlier procedure. Technometrics. 1983;25:165‐172.

[40] R Core Team . R: a language and environment for statistical computing. Vienna, Austria: R Foundation for Statistical Computing; 2013.

[41] Dancho M , Vaughan D . Anomalize: tidy anomaly detection. R package version 0.2.0. 2019 https://CRAN.R-project.org/package=anomalize

[42] Van Rossum, G , & Drake, FL . The python language reference manual. Network Theory Ltd., 2011.

[43] Wickham H . Ggplot2: elegant graphics for data analysis. New York: Springer-Verlag; 2016.

[44] Ayers JW , Caputi TL , Nebeker C , Dredze M . Don't quote me: reverse identification of research participants in social media studies. NPJ Digit Med. 2018;1:30. 31304312 10.1038/s41746-018-0036-2 PMC6550214

[45] Zook M , Barocas S , Crawford K , et al. Ten simple rules for responsible big data research. PLOS Comput Biol. 2017;13(3): e1005399 10.1371/journal.pcbi.1005399 28358831 PMC5373508

[46] Pollock C . TexasSenate tentatively approves fetal remains bill. The Texas Tribune. https://www.texastribune.org/2017/03/29/texas-senate-tentatively-approves-fetal-remains-bill/. Published March 29, 2017. Last Accessed October 18, 2019.

[47] Donahue JG , Kieke BA , King JP , et al. Inactivated influenza vaccine and spontaneous abortion in the vaccine safety datalink in 2012–13, 2013–14, and 2014–15. Vaccine. 2019;37:6673‐6681. 31540812 10.1016/j.vaccine.2019.09.035 PMC6906603

[48] Medley N , Vogel JP , Care A , Alfirevic Z . Interventions during pregnancy to prevent preterm birth: an overview of cochrane systematic reviews. Cochrane Database Syst Rev. 2018;11:CD012505. 30480756 10.1002/14651858.CD012505.pub2 PMC6516886

[49] March of Dimes . Premature birth report cards. https://www.marchofdimes.org/mission/prematurity-reportcard-tv.aspx. Accessed November 30, 2019.

[50] Allen R , O'Brien BM . Uses of Misoprostol in obstetrics and gynecology. Rev Obstet Gynecol. 2009;2:159‐168. 19826573 PMC2760893

[51] Hripcsak G , Rothschild AS . Agreement, the f‐measure, and reliability in information retrieval. J Am Med Inform Assoc. 2005;12:296‐298. 15684123 10.1197/jamia.M1733 PMC1090460

[52] Broniatowski DA , Jamison AM , Qi S , et al. Weaponized health communication: twitter bots and russian trolls amplify the vaccine debate. Am J Public Health. 2018;108:1378‐1384. 30138075 10.2105/AJPH.2018.304567 PMC6137759

[53] Chang J , Rosenn I , Backstrom L , Marlow C . ePluribus: ethnicity on social networks In: Procedinngs of the fourth international conference on weblogs and social media. Washington, DC: Association for the Advancement of Artificial Intelligence (AAAI); 2010:18‐25.

[54] Rao D , Paul MJ , Fink C , Yarowsky D , Oates T , Coppersmith G . Hierarchical bayesian models for latent attribute detection in social media In: Proceedings of the Fifth International Conference on Weblogs and Social Media. Menlo Park, CA: ACM; 2011:598‐601.

[55] Pennacchiotti M , Popescu A‐M . A machine learning approach to twitter user classification In: Proceedings of the fifth international AAAI conference on weblogs and social media. Menlo Park, CA: Association for the Advancement of Artificial Intelligence (AAAI); 2011:281‐288.

[56] Bergsma S , Dredze M , Van Durme B , Wilson T , Yarowsky D . Broadly improving user classification via communication‐based name and location clustering on twitter. In Proceedings of the 2013 Conference of the North American Chapter of the Association for Computational Linguistics: Human Language Technologies. 2013;1010‐1019.

[57] Liu W , Ruths D . Using first names as features for gender inference in Twitter Analyzing Microtext. Palo Alto, CA: AAAI Press; 2013.

[58] Dimitriadis I , Batsis M , Petrozza JC , Souter I . Racial disparities in fertility care: an analysis of 4537 intrauterine insemination cycles. J Racial Ethn Health Disparities. 2017;4(2):169‐177. 26983623 10.1007/s40615-016-0215-2

[59] Brandzel S , Chang E , Tuzzio L , et al. Latina and Black/African American women's perspectives on cancer screening and cancer screening reminders. J Racial Ethn Health Disparities. 2017;4(5):1000‐1008. 10.1007/s40615-016-0304-2 PMC5436953 27864808

[60] Bellhouse C , Temple‐Smith M , Watson S , Bilardi J . “The loss was traumatic… some healthcare providers added to that”: Women's experiences of miscarriage. Women and Birth. 2019;32:137‐146. 30153984 10.1016/j.wombi.2018.06.006

[61] Evans L , Lloyd D , Considine R , Hancock L . Contrasting views of staff and patients regarding psychosocial care for Australian women who miscarry: a hospital based study. Aust N Z J Obstet Gynaecol. 2002;42:155‐160. 12069141 10.1111/j.0004-8666.2002.00155.x

